# Influence of strength and power training on the rate of force development, peak force and functional mobility in elderly people with Parkinson's disease: a randomized controlled trial

**DOI:** 10.3389/fneur.2025.1465848

**Published:** 2025-01-27

**Authors:** Késia Maísa do Amaral Felipe, Patrícia de Aguiar Yamada, Tamires Meneghetti, Flávia Roberta Faganello-Navega

**Affiliations:** ^1^Institute of Biosciences, Graduate Program in Human Development and Technologies, Paulista State University UNESP, Rio Claro, SP, Brazil; ^2^Anhanguera Faculty of Jundiaí, Jundiaí, SP, Brazil; ^3^School of Philosophy and Sciences, Department of Physiotherapy and Occupational Therapy, Paulista State University UNESP, Marília, SP, Brazil

**Keywords:** elderly people, Parkinson's disease, muscle strength, muscle power, lower limb

## Abstract

**Objective:**

To analyze the influence of lower limb muscle strength and muscle power training on rate of force development (RFD) and peak force (PF) in elderly people with Parkinson's disease (PD), as well as the effect of these training sessions influence on the functional mobility (FM) of this population.

**Methods:**

This was a randomized controlled clinical trial and registered on the virtual platform for registration of experimental and non-experimental studies “Registro Brasileiro de Ensaios Clínicos (ReBEC)”. Thirty four elderly people of both genders without and with PD were divided into four groups: strength training control (GSC, *n* = 8); potency training control (GPC), *n* = 9; subjects with PD submitted to strength training (GSPD, *n* = 8); subjects with PD submitted to potency training (GPPD, *n* = 9). GSC and GPC consisted of with no history of neurological diseases. PF and RFD in the first 50 and 200 milliseconds (ms) were determined. FM was also assessed using the following tests: gait speed test (GS), Timed Up and Go (TUG), Short Physical Performance Battery (SPPB), Unified Parkinson's Disease Rating Scale (UPDRS); parallel feet on a force platform. Next, the participants underwent lower limbs muscle strength or muscle power training for eight consecutive weeks twice a week and were then re-evaluated.

**Results:**

The repeated measures ANOVA test showed a significant difference for PF, RFD and FM regardless of training type.

**Conclusion:**

The proposed muscle strength and muscle power training protocols influenced the increase in RFD, PF and FM of all participants; however, the increase in RFD in the first 200 ms was more pronounced in the groups submitted to power training and the increase in PF was more pronounced in the groups submitted to strength training.

## 1 Introduction

Future projections estimate an increase in the number of elderly people over the coming decades. With the increase in this population, it is expected that the number of chronic diseases associated with aging will also increase, one of these conditions being Parkinson's disease (PD) (1–3).

Among the various physical valence conditions of patients with PD, there is a reduction in muscle potency and power (4). Power and muscle potency are related to the capacity to produce maximum force (peak force) and to the rate of force development (RFD) (5–8). Paying attention to such variables is extremely important, since the production of maximum strength is related to the best functional capacity, which guarantees independence in performing activities of daily living (ADL); (9) this capacity, however, was progressively reduced in patients with PD (10). RFD is also reduced in this population since the activation of central motor areas at the beginning of a movement is not completely effective (11, 12). RFD is the production of rapid force at the beginning of a movement, which allows the resumption of stability in situations of postural oscillations, preventing falls (13).

In addition, studies indicate that the reduction in strength and muscle potency is also related to the decline in functional mobility (FM), predisposing these individuals to great difficulty in performing ADL and to a higher risk of falls, generating potentially harmful consequences to the quality of life of this population (5, 6).

Studies point to the effectiveness of strength training and muscle potency in elderly people with PD in improving the activation and synchronization of motor units, as well as promoting central and peripheral adaptations, positively influencing muscle functions (RFD and maximum force production, for example) and FM (14). However, these studies exclusively analyzed one or another type of training, that is, strength or muscle potency (7, 15–18). Therefore, we are currently in a phase where it is necessary to differentiate the types of training so that it will possible to identify which rehabilitation protocol can be more effective for the population with PD. This is only possible by comparing the effect of the two types of training (potency × power) on variables such as RSD, peak force (PF), and, consequently, FM. Scientific analysis provides clinical applicability to achieve better results in the rehabilitation of elderly people with PD, an extremely important objective since PD is the second most common neurodegenerative disease affecting the older population (19).

According to the above considerations, the primary objective of the present study was to analyze the influence of strength training (ST) and potency (PT) of the lower limbs on the RFD and PF of elderly people with PD, with the secondary objective of analyzing the influence of these trainings on the FM of this population.

We hypothesized that both ST and PT are effective in improving the variables analyzed. However, we believe that, due to the bradykinesia of elderly people with PD, a training that involves greater speed to perform the movement (potency) may be more effective for increasing RFD since this variable involves the rapid recruitment of muscle fibers.

## 2 Methods

### 2.1 Study design

This was a randomized controlled clinical trial approved by the local Ethics Committee under number 2.235.715 and registered on the virtual platform for registration of experimental and non-experimental studies “Registro Brasileiro de Ensaios Clínicos (ReBEC)”, under number RBR−2zr6fg. All participants signed an informed consent form.

The study was carried out in the physiotherapy laboratories and in the *Centro de Estudos da Educação e Saúde* (CEES) of the Faculty of Philosophy and Sciences of UNESP in *Mar*í*lia*, SP.

### 2.2 Participants

Participants were recruited through postings on social networks, leafleting on the streets and at bus terminals, doctors' offices, and basic health units. Elderly people with PD were also recruited at the CEES, where a group of individuals with PD is assisted by physical therapists.

The sample consisted of 34 elderly people (over 60 years old) of both sexes with and without PD, who were divided randomly into four groups: strength training control (GSC, *n* = 8); potency training control (GPC, *n* = 9); subjects with PD for strength training (GSPD, *n* = 8); subjects with PD for potency training (GPPD, *n* = 9). A block randomization was used. Elderly people with no history of neurological diseases were included in GSC and GPC and elderly people with idiopathic PD, classified as stages I to III of the Hoehn and Yahr scale, were included in GSPD and GPPD (20). All collection procedures were performed in the “on” phase of PD drugs.

Exclusion criteria for the PD groups were: being in the pharmacological adaptation phase, and having freezing. Exclusion criteria for all participants were: not performing independent gait, having pain, fracture, or severe soft tissue injury in the 6 months prior to the study, and having a history of uncontrolled cardiovascular, respiratory or metabolic changes, as well as cognitive alterations (21).

### 2.3 Procedures

Data were collected on 2 days with a 48 h break between them, always during the same period. On the first day the participants were instructed about the objectives and procedures of the study, and answered questions about the number of falls during the last 12 months, presence of other comorbidities, duration of the disease (for the participants with PD) and medications in use and schedules. Body weight and height were also measured. Subsequently, they were submitted to FM assessment using the following instruments: Gait Speed Test (GS), Timed Up and Go (TUG) and Short Physical Performance Battery (SPPB). Individuals in the STDP and PTDP groups were also assessed using part III of the Unified Parkinson's Disease Rating Scale (UPDRS).

Also on the first day, after FM assessment, the participants were submitted to familiarization with muscle function (MF) assessment, i.e., determination of PF and RFD of the lower limbs. RFD was then evaluated on the second day (48 h later). This break between assessment days was adopted in order to avoid fatigue of the subject's lower limbs when performing the RFD assessment.

Finally, 2 days a week were scheduled with each participant with a break of at least 48 h between days for the execution of ST or PT for the lower limbs for eight consecutive weeks. After completion of all sessions, the participants were reassessed.

### 2.4 Lower limb MF assessment

MF was assessed by analyzing RFD in the first 50 and 200 ms and PF. For this procedure, a horizontal leg press machine was used with a load cell of 500lb-F (2200N) capacity which was attached with steel cables (Myovideo, Noraxon, Arizona, USA) (22, 23). The measured variables were later processed using specific routines developed in a Matlab environment (Mathworks^®^). The participant was positioned sitting and leaning against the equipment, with arms crossed on the chest, with the ankle positioned at 0° flexion (neutral position) and the knee at 60° flexion (22, 23).

The protocol consisted of performing three submaximal voluntary isometric contractions, which were maintained for 5 s with a 30 s break between them. Before performing these contractions, the volunteer received the following instructions: “When I say ‘go', you will push this platform with your feet, but without putting all your strength into this task, and will continue pushing until I say ‘stop”'. After the submaximal contractions, the volunteers were allowed to rest for 5 min and data collection was then started. For the collection, the volunteers performed three maximum voluntary isometric contractions, which were maintained for 5 s with a 30 s break between them. During each contraction, the participants were instructed to push as hard and fast as possible the platform on which the feet were supported. Before performing these contractions, the volunteer received the following instructions: “When I say ‘go', you will push this platform with your feet as hard and as fast as possible, and you will keep pushing until I say ‘stop”' (24).

### 2.5 FM assessment

The assessment of habitual GS, which is capable of detecting changes in FM, was performed using the 10 m walk test. For this assessment, the participants walked at their normal speed to cover a distance of 12.4 m, with first and last 1. m being disregarded to eliminate component acceleration and deceleration. This test was performed three times and the arithmetic mean of the values obtained in the three trials was then calculated (25–27).

The TUG consists of measuring the time taken by the participant to start from the sitting position, to get up from the chair, walk three meters, turn around and return to the sitting position. This is a test that can evaluate the mobility of the individual who performs it, with the highest time values epresenting less functionality; 10 s is the time considered normal for healthy individuals (28).

The SPPB is an instrument widely used to assess an individual's functional capacity through tests of static balance, gait speed and lower limb strength (29).

The UPDRS—part III is a scale widely used to assess the motor signs of PD and to monitor disease progression. It consists of 14 items, with the highest score indicating greater impairment (30).

### 2.6 Training protocol

The ST and PT protocols were performed twice a week for 8 weeks, each session lasting 60 min, with a minimum of a 48-h break between sessions. Blood pressure and heart rate were measured before each session. The initial 10 min of the session were devoted to warming up by walking on flat ground. The mechanotherapy equipment used in both types of training were: leg press, extension chair, flexor chair, abductor chair, and adductor chair.

The exercises were performed in three sets of 10 repetitions with an interval of 3 min between sets and between exercises (31, 32). The load value of a maximum repetition (MR) was measured before the beginning of the first training session. MR is defined as the maximum load that can be raised once throughout the entire range of motion without compensation. Subsequently, MR was recalculated every 2 weeks during training so that each load could be readjusted based on the last MR calculation (33).

For the ST groups, training was at 70% of 1 MR during the first 2 weeks, at 80% of 1 MR from the third to the fifth week, and at 90% of 1MR over the subsequent weeks. The speed of movement execution was 2 s in both the concentric and the eccentric phase, for a total of 4 s (34, 35).

For the PT groups, training was at 40% of 1 MR in the first 2 weeks, at 50% of 1 MR from the third to the fifth week, and at 60% of 1 MR over the subsequent weeks. The speed of movement execution was as fast as possible in the concentric phase and 2 s in the eccentric phase (6, 36).

### 2.7 Data analysis

For the analysis of MF data, the highest value obtained among the three maximum voluntary isometric contractions was considered (37). These data were processed using specific routines developed in a Matlab environment (Mathworks^®^), with the analysis of PF (Nm.kg^−1^) and RFD (Nm.s^−1^.kg^−1^) of the hip and knee extensor muscles in the first 50 and 200 ms. This value was normalized by the body mass of each participant. To determine the RFD, the onset of force was defined as the point at which it exceeded 5% of the peak (24, 38). RFD was calculated using the following equation:


$$\begin{array}{l} RFD=\frac{Torque\;n=100-\;Torque\;n=1}{10samples/200Hz} \end{array}$$

Where RFD is the rate of force development, Torque *n* = 100 is the torque value in the 100th sample, Torque *n* = 1 is the torque value in the 1st sample, 100 samples is the number of samples in the set, and 2,000 is the equipment sampling frequency.

### 2.8 Randomization and masking

**Figure 1:**
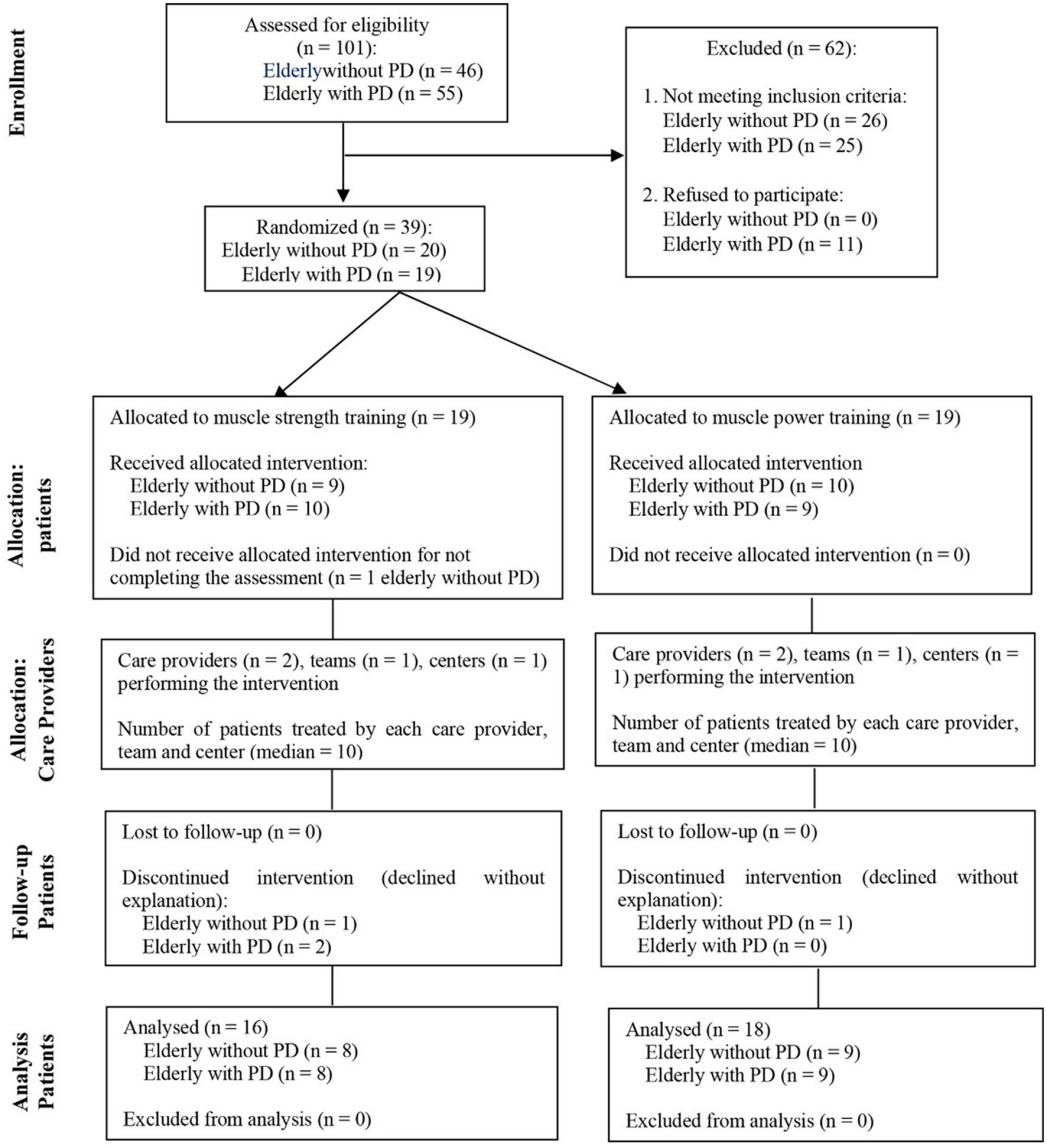
Modified CONSORT flow diagram for individual randomized controlled trials of nonpharmacologic treatments.

Block randomization was performed by this study lead author. Elderly people with no history of neurological diseases were randomly allocated to the control groups (two blocks with 10 people) while elderly people diagnosed with PD were randomly allocated to the people with the disease groups (two blocks: one with 10 people and other with nine people). Participants were allocated to groups in the order in which they registered to participate in the study. The first 10 participants enrolled were allocated to the strength training groups (both in the control group and in the PD group), and the remaining participants were allocated to the power training groups. Thirty-nine elderly people started the program, however, only 34 completed it. Figure 1 shows the modified CONSORT flow diagram for randomized controlled trials of non-pharmacological treatment (38).

The elderly people were aware that they were participating in muscle training, but no further details were disclosed.

### 2.9 Statistical analysis

The sample size estimates were based on the RDF values at the first 50 ms and 200 ms obtained before training. These variables were selected as key components of the primary outcome of the study (MF assessment). Sample size calculations were performed using G^*^Power (version 3.1.9.7) with an *a priori* approach for repeated-measures ANOVA with between-subject factors, considering four groups and two repeated measures per group. The analysis was conducted using the data from the first five participants in the study. For the RDF at 200 ms, the analysis assumed an effect size of 5.74, α = 0.05, and power (1-β) of 0.95, resulting in a required sample size of eight participants. For the RDF at 50 ms, with an effect size of 2.94, α = 0.05, and power of 0.95, eight participants were also required. Both analyses yielded an actual power of 0.99, ensuring high sensitivity for detecting significant effects.

The data of the tests performed are reported as mean and standard deviation.

Data normality was determined by the Kolmogorov-Smirnov test. To compare the groups with respect to sample characterization data, the *t*-test was used for the variables referring only to the Parkinson's groups (HeY, levodopa dosage and time of diagnosis). One Way Anova was used for the other non-categorical variables referring to all groups, and the chi-square test was used for categorical variables.

For the data referring to the RFD, PF and FM tests, the Two-Way Repeated Measures ANOVA test with Bonferroni's *Post-hoc* test was used to compare both intragroup and intergroup assessments and reassessments. The level of significance adopted was *p* < 0.05. Partial eta square was used to measure the size of the effect, with 0.01, 0.06, and 0.14 or above being considered small, medium and large, respectively (39). All analyses were performed using the PASW statistics 18.0 software. ^®^ (SPSS).

## 3 Results

No relevant adverse events occurred during the study.

Table 1 shows the homogeneous characterization of the volunteers.

**Table 1:** Sample characterization.

**Characteristics**	**GSC (*n* = 8)**	**GSPD (*n* = 8)**	**GPC (*n* = 9)**	**GPPD (*n* = 9)**
Male/female (*n*)	2/6	3/5	3/6	4/5
Age (years)	68.13 (5.28)	72.75 (6.91)	67.67 (5.64)	71.78 (8.92)
Weight (kg)	79.4 (16.59)	71.41 (8.99)	81.93 (15.46)	70.89 (15.89)
Height (m)	1.60 (0.06)	1.60 (0.08)	1.61 (0.11)	1.61 (0.12)
BMI (kg/m^2^)	31.24 (6.21)	27.70 (2.06)	31.59 (5.87)	27.16 (4.69)
HeY		2.25 (0.56)		2.39 (0.61)
Levodopa Dosage (mg/day)		425 (227.76)		311.11 (136.99)
Time of diagnosis (years)		6.25 (4.05)		6.44 (3.06)

Values are reported as mean and standard deviation.

BMI, body mass index; GSC, strength training control group; GSPD, group of elderly people with Parkinson's disease submitted to strength training; GPC, power training control group; GPPD, group of elderly people with Parkinson's disease submitted to power training; HeY, Hoehn and Yahr scale.

### 3.1 MF

Table 2 shows the mean and standard deviation values of PF (N), RFD in the first 50 ms (N) and RFD in the first 200 ms (N) during the evaluation and reevaluation periods for both groups.

**Table 2:** Mean and standard deviation values; repeated measures Anova *F, P-*values and effect size (partial square eta) for PF, RFD in the first 50 ms and RFD in the first 200 ms.

	**PF(N)**			**Evaluations effects**	**RFD 0-50ms (N)**			**RFD 0-200ms (N)**		
		**Mean** ±**SD**	**Groups effects**		**Mean** ±**SD**	**Groups effects**	**Evaluations effects**	**Mean** ±**SD**	**Groups effects**	**Evaluations effects**
GSC	E	11.6 ± 7.9			4.8 ± 2.2			11.3 ± 6.4		
	RE	18.9 ± 6.4			10.0 ± 6.3			18.3 ± 9.0		
GSPD	E	13.3 ± 5.2	*F* = 2.18	*F* = 24.49	9.0 ± 6.2	*F* = 0.57	*F* = 9.19	10.4 ± 5.5	*F* = 6.04	*F* = 26.81
	RE	22.8 ± 10.0	*P* = 0.20	*P* = 0.001^*^	11.5 ± 5.8	*P* = 0.65	*P* = 0.019^*^	15.3 ± 10.5	*P* = 0.04^*^	*P* = 0.001^*^
GPC	E	12.1 ± 7.8	ES = 0.16	ES = 0.79	3.5 ± 1.5	ES = 0.09	ES = 0.56	8.4 ± 4.0	ES = 0.20	ES = 0.79
	RE	22.6 ± 8.6			13.6 ± 11.9		19.4 ± 6.4		
GPPD	E	9.8 ± 6.2			4.9 ± 4.3			4.8 ± 2.3		
	RE	12.6 ± 5.9			7.5 ± 5.9			12.1 ± 5.5		

PF, peak force; RFD, rate of force development; GSC, strength training control group; GSPD, group of elderly people with PD submitted to strength training; GPC, Power training control group; GPPD, group of elderly people with PD submitted to power training; E, Evaluation; RE, Reevaluation; ES, effect size.

^*^Denotes a significant difference (*p* < 0.005).

Statistical analysis showed the effect of assessments (assessment and reassessment) for PF, and RFD in the first 50 ms. There was no effect of groups or interaction between groups and assessments for these variables. For RFD in the first 200 ms, there was an effect of groups and assessments, but there was no effect of interaction between groups and assessments. Table 2 presents the *F-* and *P*-values for each test.

### 3.2 FM

Table 3 shows the mean and standard deviation values of the GS, TUG, SPPB and UPDRS tests during the assessment and reassessment periods of the groups.

**Table 3:** Mean and standard deviation values; repeated measures Anova, *F, P-*values and effect size (partial square eta) for GS, TUG, SPPB and UPDRS test.

	**GS (m/s)**				**TUG (s)**			**SPPB (score)**			**UPDRS (score)**		
		**Mean** ±**SD**	**Groups effects**	**Evaluations effects**	**Mean** ±**SD**	**Groups effects**	**Evaluations effects**	**Mean** ±**SD**	**Groups effects**	**Evaluations effects**	**Mean** ±**SD**	**Groups effects**	**Evaluations effects**
GSC	E	1.1 ± 0.2			11.3 ± 2.7			10.7 ± 1.2					
	RE	1.2 ± 0.2			9.8 ± 1.8			11.6 ± 0.7					
GSPD	E	1.0 ± 0.1			12.8 ± 1.7			9.2 ± 1.5			30.7 ± 11.7		
	RE	1.2 ± 0.2	*F* = 1.94	*F* = 41.0	12.2 ± 1.9	*F* = 1.35	*F* = 4.63	10.1 ± 1.3	*F* = 2.26	*F* = 15.90	25.1 ± 8.7	*F* = 0.04	*F* = 14.0
GPC	E	1.2 ± 0.2	*P* = 0.24	*P* = 0.00^*^	11.4 ± 2.4	*P* = 0.35	*P* = 0.06	10.2 ± 1.5	*P* = 0.19	*P* = 0.005^*^		*P* = 0.83	*P* = 0.007^*^
	RE	1.2 ± 0.1	ES = 0.19	ES = 0.85	10.4 ± 1.3	ES = 0.34	ES = 0.39	11.4 ± 0.9	ES = 0.31	ES = 0.69		ES = 0.07	ES = 0.06
GPPD	E	0.9 ± 0.1			15.6 ± 4.0			8.4 ± 2.2			30.2 ± 12.6		
	RE	1.0 ± 0.1			15.6 ± 6.3			9.2 ± 2.0			25.7 ± 10.2		

GS, gait speed; TUG, timed up and go; SPPB, short physical performance battery; UPDRS, Unified Parkinson's Disease Rating Scale; GSC, strength training control group; GSPD, group of elderly people with PD submitted to strength training; GPC, Power training control group; GPPD, group of elderly people with PD submitted to power training; E, Evaluation; RE, Reevaluation; ES, effect size.

^*^Denotes a significant difference (*p* < 0.005).

For the GS, SPPB and UPDRS tests, statistical analysis showed that there was an effect of assessments (assessment and reassessment) and that there was no effect of groups or interaction between groups and assessments. The analysis showed no significant difference in TUG. Table 3 presents the *F*- and *P-*values of each test.

## 4 Discussion

The results of the present study regarding both protocols showed significant improvement for PF, RFD, GS test, SPPB and UPDRS in elderly people with and without PD.

PF is reduced in patients with PD and the improvement of this variable may result in functional gains (40). The increase in PF after a resistance training protocol is due to changes in neural command and activation of the PF muscle generated by the training itself (41). In addition, our findings corroborate those of Bologna et al. (41) who stated that resistance training improves PF and the clinical symptoms of PD, as we also observed based on the reduction in the UPDRS-III score. However, according to Maffiuletti et al. (42), RFD is even more sensitive than PF for the detection of changes in neuromuscular function and seems to have a greater influence on the performance of ADL. It is worth mentioning that, according to the cited authors, RFD seems to be related more to muscle potency than to the force (42). Thus, although both types of training in the present study were able to increase this variable, the increases in RFD in the first 200 ms were more pronounced in the groups that trained potency when compared to the groups that trained strength: GSC increased by 62.2% from 11.31 to 18.35 N after the training period; in GSPD it increased by 47.7% from 10.40 to 15.31 N, while in GPC it increased by 130% from 8.45 to 19.45 N, and in GPPD it increased by 149.5% from 4.86 to 12.13 N.

The mean values obtained revealed that, while the groups of individuals with PD who trained potency showed a more pronounced improvement in RFD, the groups that trained strength showed more pronounced improvement in PF, confirming the principle of training specificity. The type of training generates morphofunctional changes through muscle plasticity, defined as the ability of skeletal muscle to change its structural and functional properties according to the stimuli received, enabling changes in the types of fibers that form a given musculature (43).

In the present study, RFD was analyzed at two time points, i.e., in the first 50 ms and at 200 ms. It is worth mentioning that in the most initial phase of muscle contraction, here represented by the analysis of the first 50 ms, there is greater neural influence for the contraction to occur, while in the later phase of the beginning of the contraction, represented here by the 200 ms, the greatest contribution is related to the intrinsic properties of the muscle (42). All groups in the present study were able to increase PF and RFD in the first 50 and 200 ms after 8 weeks regardless of the type of training, thus suggesting that both the ST and PT protocol positively influenced the improvement in the firing of motor units demonstrated by the increase in RFD in the first 50 ms, as well as the improvement in force production, demonstrated by the increase in the peak RFD in the first 200 ms which, according to Maffiuletti et al. may contribute to the reduction of falls (42).

In contrast to our findings, Schlenstedt et al. (7) found no improvement in PF after 8 weeks of lower limb resistance training in Parkinson's patients, and some improvement in PF occurred only on the less affected side. In the present study, we found a significant improvement in PF and RFD by evaluating these variables bilaterally, i.e., by testing the lower limbs simultaneously, since in most ADLs the lower limbs are recruited at the same time. Thus, so we cannot say that the improvement in results obtained by us was due to the improvement of one or the other limb, although it should be pointed out that the methodology used by the aforementioned authors differed from the one used in the present study, since the resistance applied by them was not through mechanotherapy equipment, which allows a collection environment with better control of load increment (7). The results of the cited authors also indicate a relationship between the increase in RFD and the improvement of balance in patients with PD assessed using the Fullerton Advanced Balance—FAB Scale. In our study, there was also a significant improvement in RFD and, considering that tasks involving balance make up the SPPB and UPDRS tests that showed significant improvements in our study, we can say that our findings corroborate the findings of the aforementioned authors in this regard (7).

The improvement in MF observed in the present study through the increase in PF and RFD in the first 50 and 200 ms is fundamental by inferring that both muscle strength and potency training should be included in the treatment of patients with PD, since these variables directly influence the individual's ability to perform tasks such as sitting/standing up and going up/down stairs, among other functional activities (43, 44). Thus, tests that assess FM were analyzed in the present study, demonstrating the influence of the training proposed by us on this variable.

According to Dommershuijsen et al. (45), decreased GS is one of the first signs of aging, considered a general health marker strongly associated with the risk of mortality (45). Therefore, we suggest the relevance of the two training models proposed in our study since statistical analysis showed a significant increase in GS between assessment and reassessment, regardless of the group. We can relate the increase in GS to the gain in muscle strength demonstrated by the increase in PF and RFD. Huang et al. (46) suggested that the reduction in walking speed may be partially explained by the peripheral component of knee extensor strength. The loss of peripheral strength is related to failures in neuromuscular transmission, at the neuromuscular junction or in the musculature itself (46). Similar results were obtained by Allen et al. (47) who showed that muscle strength was a significant determinant of walking speed in PD patients, even after adjusting for the UPDRS motor score (47). A possible explanation for the relationship between strength and movement speed in PD patients was given by David et al. (48) who suggested that the improvement in movement speed resulting from progressive resistance training is due to the fact that this type of training restores some properties of the EMG muscle activation pattern and improves the strength of the trained muscles. Together, changes in muscle activation and muscle strength are significantly associated with improved slowness of movement (48).

Still regarding the GS results, a relevant factor is that the GSPD, which in the pre-training assessment had a speed of 1.07 m/s, started to walk at a speed > 1.22 m/s after 8 weeks of training, a speed considered suitable for the elderly gait, and that the National Traffic Department (DENATRAN) uses to determine the traffic light timing for pedestrians to cross the street safely (49). This increase in GS represents a reduction in potentially harmful risks such as being run over, resulting in a safer community life.

Regarding SPPB, our results were similar to those of GS, that is, regardless of the group, elderly people with or without PD who performed strength or potency training performed better the SPPB after the proposed training. A study carried out with the elderly also found an improvement in the performance of SPPB after resistance training at low or high speed (50). Taking into account that SPPB can be used as a valid tool to assess not only mobility, but also the risk of falling in the elderly (51–53), we can suggest that the proposed training was able to reduce the risk of falls in elderly people with or without PD. However, there are no studies in the literature that have verified whether the SPPB score can also predict falls in elderly people with PD. Thus, we suggest that studies be carried out in order to determine if the SPPB score can also predict the incidence of falls in elderly people with PD. The literature has shown that different treatments proposed for individuals with Parkinson's, such as treadmill training and physical therapy interventions, can improve the score of this assessment (54, 55). Antônio et al. (15) found improvement in the sit/stand test (a test included in the SPPB) after 12 weeks of strengthening in individuals with PD, in addition to a negative correlation of this test with the measure of functional independence (MFI), that is, the shorter the time to perform the test, the higher the MFI score, thus suggesting that the greater the lower limbs strength, the greater the functional independence (15).

Regarding the TUG, the findings of the present study show that this was the only test performed outside the force platform that did not show a statistically significant improvement. Schenkman et al. (56) suggest that the extra time to perform the TUG is more clearly evidenced in patients with higher disease levels (H&Y stage 3; UPSRS-III score = 45.5–60). In our study, patients had an average score of 2.25 and 2.39 in GSPD and GPPD, respectively, on the H&Y scale, with patients in stage 3 of the disease representing the minority of the sample, a result that may have occurred because we did not include patients who reported freezing, which is most often found in the most advanced stages of the disease. Furthermore, the average was 25.12 and 25.77 points in GSPD and GPPD in UPDRS-III after 8 weeks of training (56). The present TUG result agrees with the findings of Peterson et al. (57) who suggested that measures effective in increasing gait speed may not be efficient for tasks that require specific changes of turn (58). It is worth noting that difficulties in turning during gait are especially common among individuals with PD, reducing functional independence (58). According to Mancini et al. (59), specific tasks require specific training.

According to Tanji et al. (60), both the SPPB and the TUG are significantly correlated with measures of disability and disease severity; however, the TUG assesses the performance of several tasks carried out together (getting up from a chair, walking, turning around and sitting), with greater complexity, while the SPPB, as well as other tests, evaluate the tasks separately. This may justify the fact that the TUG was the only test used in the present study that did not show significant improvement (60).

The UPDRS has been used to better assess patients with PD. Schenkman et al. (56) correlated the UPDRS with other functional tests, demonstrating that the reduction in the scale score reflects better functionality in patients with PD. The authors also pointed out that patients with scores between 30.5 and 45 on the UPDRS-part III had a score on the Continuous Scale Physical Functional Performance Test (CS-PFP) that indicated a transition from independence to functional dependence (56). In the present study, GSPD that had a score of 30.75 went on to show 25.13, and GPPD that had a score of 30.22 went on to show 25.78 points after 8 weeks of training. The significant improvement in the scale score (part III) of both groups demonstrates that both ST and PT reduce the severity of the patients' motor signs and symptoms, distancing them from the levels of functional dependence.

In the present study, the PD groups behaved similarly to the control groups regardless of the type of training, that is, they showed the same muscle and FM gains.

The results of the present study demonstrate that, since PD is a progressive disease, ST and PT of the lower limbs should be included in the treatment of patients with the disease since these types of training allowed this population to achieve results similar to those for older subjects without the disease, indicating that strength training and muscle potency reduce the impacts of the pathology on the patients. Although training generated important gains for the groups of individuals with PD and for individuals without the disease, the evidence of lower muscle contraction capacity on the part of the older subjects with PD, represented by the lower values of RFD in the first 200 ms, demonstrated the influence of the disease on the muscular components. Furthermore, although both types of training positively influenced MFs and FM, PT seems to be more influential in improving RFD in the first 200 ms. We suggest that new studies be carried out comparing ST and PT so that more conclusive results can be obtained about the influence of these types of training on muscle functions.

### 4.1 Limitations

We should mention that the small sample size is one of the limitations of the study. This is due to the difficulty in finding patients with PD who fit the eligibility criteria, such as not having freezing and performing independent gait (without assistive devices).

## 5 Conclusion

The ST and PT protocols proposed in the present study influenced the increase in RFD, PF and FM of the participants; however PT seemed to have a greater influence on the increase in RFD in the first 200 ms, while ST seemed to have a greater influence on the increase in PF.

Considering that PD is a progressive pathology, the fact that elderly subjects with the disease showed similar responses to the control groups for all variable analyzed after 8 weeks of training, also demonstrates the effectiveness of the training performed.

## Data Availability

The original contributions presented in the study are included in the article/supplementary material, further inquiries can be directed to the corresponding author.
